# Trends in cancer incidence and mortality over three decades in Quito - Ecuador

**DOI:** 10.25100/cm.v49i1.3785

**Published:** 2018-03-30

**Authors:** Fabián Corral Cordero, Patricia Cueva Ayala, José Yépez Maldonado, Wilmer Tarupi Montenegro

**Affiliations:** 1 Director Honorífico de los Registros de Cáncer del Ecuador - Fundador del Registro Nacional de Tumores. Quito, Ecuador; 2 Registro Nacional de Tumores, Quito, Ecuador.; 3 Sociedad de Lucha contra el Cáncer. SOLCA Quito, Ecuador; 4 Facultad de Ciencias de la Salud, Universidad Tecnológica Equinoccial, Quito Ecuador.

**Keywords:** Incidence, mortality, cancer, Ecuador, Latin America, Incidencia, mortalidad, cáncer, Ecuador, América latina

## Abstract

**Introduction:**

The National Registry of Tumors has collected, processed, analyzed and regularly disseminated information on new cases of cancer diagnosed in the city of Quito, Ecuador over the last three decades.

**Aim:**

This article analyzed the trend of cancer incidence and mortality rates for the period 1985-2013.

**Methods:**

Incidence and mortality rates standardized by age were estimated by the direct method, using the world standard population. Analysis of the time trends, from selected locations, the joinpoint regression was used.

**Results:**

A decrease in the incidence and mortality rates of cervical and stomach cancers were documented. There was an increase in breast and colorectal cancer rates. The increase of the incidence rate of thyroid cancer in women was notorious. Lung cancer also increased in women while in men their values remained stable.

**Conclusion:**

There are important variations in the evolution of cancer in Quito; the information presented is an instrument for monitoring and evaluating the interventions that are developed in the Country.

## Resumen

**Introducción:**

El Registro Nacional de Tumores ha recolectado, procesado, analizado y divulgado regularmente la información de los casos nuevos de cáncer diagnosticados en la ciudad de Quito, Ecuador durante las últimas tres décadas.

**Objetivo:**

Analizar la tendencia de las tasas de incidencia y mortalidad por cáncer durante el periodo 1985-2013.

**Métodos:**

Se estimaron las tasas de incidencia y mortalidad estandarizadas por edad a través del método directo, utilizando la población estándar mundial. Para el análisis de la tendencia de las tasas, de localizaciones seleccionadas, se utilizó la regresión joinpoint.

**Resultados:**

Se documentó un descenso de las tasas de incidencia y mortalidad de los tipos de cáncer de cuello uterino y estómago. Existe incremento de las tasas de cánceres de mama y colon-recto. Es notorio el crecimiento de la tasa de incidencia de cáncer de tiroides en mujeres. El cáncer de pulmón se incrementó en las mujeres en tanto que en los varones sus valores se mantuvieron estables.

**Conclusión:**

Se evidencian importantes variaciones en la evolución del cáncer en Quito, la información presentada constituye un instrumento para el seguimiento y evaluación de las intervenciones que se desarrollen.

## Introduction

The experience and consolidation of cancer population registries had their beginning in Hamburg, Germany in 1929 ^1^. Its greatest impulse comes from the Conference of Copenhagen which took place in 1946, and recommended the establishment of Cancer Registries Worldwide. In 1966 the International Association of Cancer Registries (IACR) was created as part of the International Agency for Research on Cancer (IARC). This entity brings together, supports and sets the guidelines for the development and implementation of cancer registries in humans across the world.

In Latin America, the first cancer registry appeared in the 1950s ^2^ in Puerto Rico, and the second one in Cali, Colombia in 1962. This encouraged the consolidation of the proposal in Ecuador. In 1984, within the Cancer Fighting Society (SOLCA) Quito Nucleus, the National Tumor Registry (NTR) was created ^3^. Since its establishment it has collected, processed, analyzed and regularly disseminated information on new cases of cancer diagnosed in the city of Quito. The present study analyzes the trend of incidence and mortality of the main types of cancer in the city of Quito, from 1985 to 2013.

## Materials and Methods

The NTR registers all cases of cancer diagnosed in the city of Quito using a methodology which is adopted internationally (IACR). The information is obtained through an active process, in which a group of technicians goes to the pathology, haematology and cytology laboratories of all public and private health center establishments in Quito. They review the clinical records to detect diagnosed cancer cases, and then read the clinical history or contact the treating doctor to obtain more information about the patient. This methodology guarantees that there are no duplication of cases.

The cases that never underwent microscopic examination are captured in the National Institute of Statistics and Census (INEC) through the review of "Hospital discharges" and in the "Deaths by cancer" occurred in the city of Quito, which constitute approximately 6% of new cases of cancer. A limitation of hospital discharges is not designed to adequately discriminate the multiple occasions that the same patient enters and leaves a hospital or for cancer cases that do not require hospitalization. Hospital discharges and deaths are compared with the registry database, with the aim of identifying new cases or updating the vital status of previously registered patients.

The variables that are collected are organized into three areas: identification of the patient, description of the tumor and clinical extension of the tumor. Staging is performed for the eight most frequent locations (cervix, breast, prostate, lung, colon-rectum, stomach, thyroid and lymphomas).

A Case is considered to be any invasive or *in situ* neoplasia incident in the year, with or without histological verification which occurs in the population of inhabitants in the city of Quito.

Tumors of an uncertain nature are not recorded. Since 2005, the information has been collected and processed with the CIEO3 Classification. Tumors are recorded, not patients. The definition of multiple tumors is established using the criteria defined by the IACR ^4^.

The information collected is validated through quality controls checked between registrars and resolution of doubts is consulted with the doctors of the Registry and/or the occasional support of a pathologist. At the end of each year, the validation tool is applied: IARCcrgTools 2.05, to establish the coherence of the main variables that should be a population register.

The NTR has a 4^th^ version computer system adjusted to the needs of the implemented methodology. The data is presented according to the International Classification of Diseases ICD 10, and analyzed, by sex, incidence and mortality rates standardized by age (using a world standard population and direct method). Analysis data included the following six periods: 1985-1988, 1989-1993, 1994-1998, 1999-2003, 2004-2008 and 2009-2013, in selected locations of the body. To study the trend of cancer incidence and mortality rates during the 1985-2013 period, the average annual percentage change (APC) was estimated. In describing the change, the terms '' increase '' or '' decrease '' were used when the APC was significantly different from zero (*p* <0.05); otherwise, the term "stable" was used. Significance tests were performed using the Monte Carlo permutation technique. All analysis were performed in the Joinpoint Regression Program version 4.5.0.1 of the Surveillance Research Program of the National Cancer Institute of the United States.

### Population

The city of Quito is the capital of Ecuador, it is the second largest and most populated city in the country. It is located at latitude 0 (0 ° 13'23" South), west of the Andes Mountain Range, at 2,800 meters above sea level. The extension of the city is 127 Km^2^ and its population for the year 2013, according to the Census Projections of the year 2010 was: 1,694,086 inhabitants ^5^.

**Figure 1:**
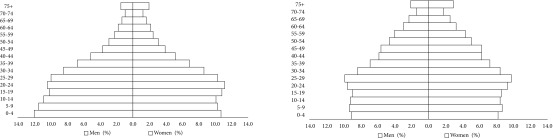
Quito Population structure by age and sex: 1985-2013.

The population of Ecuador in general and of Quito city in particular, has experienced important changes in demographic composition. Ecuador, over the last three decades, has developed censuses in the years 1982, 1990, 2001 and 2010. Through its data it can be seen that the broad base of its population pyramid decreases while the older group increases. In Quito in 1985, 54% of its population was under 25 years of age, while in 2013 this group was reduced to 44%. Those aged 65 and over accounted for 4.3% in 1985 and in 2013, 6.5% (Fig. 1). The projection of population data of the intercensal years are provided by the INEC.

The age variation and the increase in life expectancy (75 years old for females, 70 years old for males) is closely linked to the frequency of presentation and temporary tendency of cancer.

## Results

Table 1 shows the cancer standardized incidence rates per 100,000 inhabitants according to sex, main body locations and the six time periods studied. The trends of the incidence and its confidence interval, the values of the annual percentage change, and the statistical significance are also presented.

**Table 1:** Quito, Ecuador. Incidence. Standardized rates by age for locations selected by sex. 1985-2013.

Location	1985-1988		1989-1993		1994-1998		1999-2003		2004-2008		2009-2013		Trend of the incidence rate			
													Men		Women	
	♂	♀	♂	♀	♂	♀	♂	♀	♂	♀	♂	♀	APC	95% CI	APC	95% CI
Lip, oral cavity, pharynx	2.0	1.6	1.8	1.1	2.4	1.7	1.7	1.3	1.9	2.0	2.1	1.6	0.0	(-1.2; 1.3)	0.9	(-1.4; 3.2)
Esophagus	3.4	0.6	3.0	1.2	2.4	0.6	2.0	0.8	2.4	0.6	1.4	0.7	-3.1*	(-4.6; -1.5)	~	
Stomach	28.5	21.7	32.7	20.0	24.5	16.9	22.2	13.2	22.9	14.9	20.3	14.5	-1.7	(-5.3; 2.1)	-1.6*	(-2.7; -0.5)
rectal colon	7.3	8.2	7.4	9.0	8.7	8.1	8.9	8.7	11.3	10.2	13.2	11.9	2.5*	(1.7; 3.3)	1.4*	(0.6; 2.2)
Pancreas	4.4	3.6	3.7	4.2	3.8	3.5	3.2	3.1	2.8	3.6	3.2	3.9	-1.3*	(-2.3; -0.3)	0.2	(-1.2; 1.5)
Larynx	2.0	0.2	1.3	0.2	1.5	0.1	1.3	0.1	1.5	0.2	1.6	0.2	-0.1	(-1.9; 1.7)	~	
Bronchi Lung	7.9	3.3	10.1	3.7	8.3	4.8	8.4	4.4	8.0	5.8	8.0	6.4	-0.5	(-1.3; 0.4)	2.8*	(1.7; 3.9)
Cervix	-	31.0	-	32.6	-	24.8	-	19.5	-	18.6	-	18.6	-	-	-2.6*	(-3.2; -2.0)
Uterine body	-	4.8	-	5.3	-	4.5	-	4.4	-	4.4	-	5.6	-	-	0.3	(-0.6; 1.3)
Breast	-	25.4	-	26.6	-	28.4	-	31.4	-	36.8	-	38.8	-	-	1.9*	(1.4; 2.3)
Ovary	-	5.7	-	6.2	-	7.5	-	6.5	-	7.8	-	7.5	-	-	1.2*	(0.4; 2.0)
Prostate	22.7	-	23.1	-	31.7	-	43.5	-	53.5	-	62.9	-	3.8*	(2.2; 5.3)	-	-
Testicle	2.6	-	4.0	-	3.3	-	4.2	-	5.2	-	5.7	-	3.0*	(2.0; 4.1)	-	-
Bladder	4.3	2.0	5.9	1.4	5.0	1.3	5.3	1.5	5.1	2.0	6.1	2.2	0.8	(-0.3; 1.9)	1.4	(-0.2; 3.0)
Thyroid	3.1	6.3	2.1	7.6	2.2	8.7	2.4	10.5	3.9	19.6	6.6	35.0	3.6	(-0.6; 8.1)	8.5*	(5.6; 11.5)
Lymphoma	8.9	7.2	8.8	7.5	11.4	8.4	10.5	9.1	12.6	9.9	16.1	13.1	2.2*	(1.4; 3.1)	1.7	(-1.9; 5.3)
Leukemia	7.1	5.5	6.7	5.2	8.4	7.2	7.7	6.2	7.7	6.6	8.6	7.0	0.8	(-0.1; 1.8)	1.1*	(0.2: 2.1)
Melanoma	2.3	3.3	3.5	3.6	2.2	3.0	2.5	3.0	3.7	3.8	4.1	3.8	1.8*	(0.2; 3.4)	1.2	(-0.3; 2.7)
All - no melan skin	142.3	185.9	146.8	190.1	147.6	177.3	158.5	167.2	182.1	201.2	200.8	207.9	1.4*	(0.9; 2.0)	1.0*	(0.5; 1.5)

Rates x 100,000

APC: Annual percentage change. * The APC is significantly different from zero (*p* <0.05)

~ It is not possible to calculate

**Figure 2:**
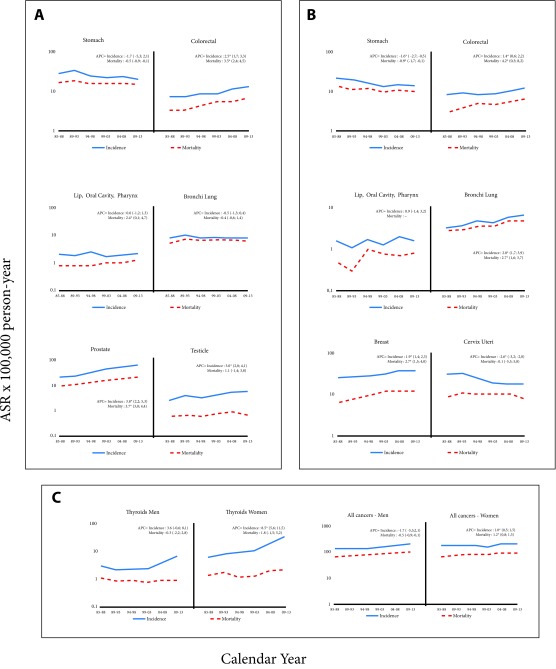
Quito, Ecuador. Rates of Cancer Incidence (Location selected). 1985-2013. **a**: Men; **b:** Women; **c:** thyroids and all cancer

In Figure 2 we can observe trends in the incidence and mortality of the main body locations. In men, a significant increase in the overall incidence rate of cancer was observed, with APC of 1.4% (Figure 2a). The stomach cancer incidence rate reached its highest point in the first two periods, although it was later overtaken by prostate cancer. Also, in women, the cancer global incidence rate increased significantly with an APC of 1% (Fig. 2b). In the first two periods, the most frequent cancer was the cervix, and in the following periods, breast cancer presented the highest rate. In the last two periods, thyroid cancer appears in second place, surpassing cervical cancer.

Table 2 shows standardized rates of cancer mortality in men and women in the six periods.

**Table 2:** Quito, Ecuador. Mortality Rates Standardized by age for selected locations by gender 1985 - 2013.

Location	1985-1988		1989-1993		1994-1998		1999-2003		2004-2008		2009-2013		Trend of mortality rate			
													Men		Women	
	♂	♀	♂	♀	♂	♀	♂	♀	♂	♀	♂	♀	APC	95% CI	APC	95% CI
Lip, oral cavity, pharynx	0.8	0.5	0.8	0.3	0.8	1.0	1.0	0.8	1.0	0.7	1.3	0.8	2.4*	(0.1; 4.7)	~	
Esophagus	2.6	0.3	2.0	0.6	1.8	0.4	1.7	0.4	1.4	0.5	1.3	0.6	-2.3*	(-3.8; -0.7)	~	
Stomach	16.7	13.8	18.4	11.5	16.2	11.6	15.8	10.2	15.9	10.6	15.2	10.0	-0.5*	(-0.9; -0.1)	-0.9*	(-1.7; -0.1)
rectal colon	3.3	2.9	3.5	3.8	4.4	4.9	5.5	4.7	5.9	5.5	7.0	6.4	3.5*	(2.4; 4.6)	4.2*	(0.3; 8.2)
Pancreas	2.7	2.6	2.8	3.3	3.1	2.7	2.5	2.5	2.5	3.3	2.6	3.1	-0.1	(-1.3; 1.1)	0.6	(-0.6; 1.9)
Larynx	1.0	0.0	0.4	0.2	0.4	0.1	0.7	0.0	1.0	0.0	0.9	0.1	~		~	
Bronchi Lung	5.2	2.8	7.2	2.9	6.7	3.5	7.0	3.6	6.7	4.6	6.2	4.9	0.4	(-0.6; 1.4)	2.7*	(1.6; 3.7)
Cervix	-	8.6	-	10.7	-	10.5	-	10.2	-	10.3	-	8.2	-	-	0.1	(-5.3; 5.8)
Uterine body	-	0.4	-	1.2	-	1.5	-	1.4	-	1.6	-	1.6	-	-	~	
Breast	-	6.5	-	7.8	-	9.6	-	12.1	-	12.4	-	12.3	-	-	2.7*	(1.5; 4.0)
Ovary	-	2.2	-	2.4	-	4.1	-	3.5	-	4.0	-	4.2	-	-	2.7*	(1.3; 4.0)
Prostate	9.3	-	10.8	-	12.7	-	16.3	-	18.7	-	1.9-1.9	-	3.7*	(3.0; 4.4)	-	-
Testicle	0.6	-	0.7	-	0.6	-	8.0	-	0.9	-	0.7	-	1.1	(-1.4; 3.8)	-	-
Bladder	1.0	0.5	1.9	0.6	1.8	0.6	2.7	1.0	2.4	0.7	2.6	1.2	5.7*	(1.0; 10.5)	2.9*	(0.1; 5.7)
Thyroid	1.1	1.4	0.9	1.7	0.9	1.2	8	1.3	0.9	2.0	0.9	2.2	0.3	(-2.2; 2.8)	1.8	(-1.5; 5.2)
Lymphoma	2.0	1.8	3.3	2.2	5.2	3.7	5.4	4.6	6.9	5.2	7.9	5.9	5.4*	(3.9; 6.9)	5.8*	(2.7; 9.0)
Leukemia	3.3	2.3	3.0	2.0	4.7	4.3	5.2	3.6	5.3	4.1	5.2	3.9	2.6*	(1.5; 3.8)	2.2	(-3.5; 8.1)
Melanoma	0.3	0.7	0.8	0.6	1.0	0.9	1.1	0.9	1.4	1.4	2.0	1.5	~		4.7*	(2.0; 7.5)
All - no melan skin	68.4	69.0	73.0	75.1	78.9	83.6	88.1	83.1	93.7	90.3	101.2	89.4	1.7*	(1.4; 2.0)	1.2*	(0.8; 1.5)

Rates x 100.000

APC: Annual percentage change. * The APC is significantly different from zero (*p* <0.05)

~ It is not possible to calculate

In men, the cancer that caused the most deaths until the period of 1994-1998 was stomach cancer. In the last three quinquennial periods, the highest mortality rate was that of prostate cancer. At the national level, the situation was different in terms of mortality, since stomach cancer until 2013 was the leading cause of death among malignant tumors. In women residing in Quito, the behavior of mortality from gastric cancer is similar to that of men with two specificities: a) the rates had lower values and b) in the period of 1999-2003, breast cancer surpassed that of the stomach as the main cause of cancer deaths.

In Figure 2 we can observe trends in the incidence and mortality of the main body locations.

Incidence rates of gastric cancer decreased in women significantly with a APC of -1.6. In men, the overall decrease was not significant. However, when applying joinpoint regression, an increase was observed in the first phase and from the period 1994-1998 until 2013, the decrease was significant. (APC -2.4, CI 95%: -3.8; -1.1). Mortality rates decreased significantly in men and women (-0.5 and -0.9, respectively).

Colon-rectal cancer had a significant upward trend in both APC, men (2.5) and women (1.4). The values were very similar for both genders. Mortality rates, in men and women, increased significantly (3.5 and 4.2, respectively)

In the 29 years of analysis, the incidence and mortality rates from lung cancer among men have remained stable. However, in women the increase was significant with a APC of 2.8 in incidence and 2.7 in mortality. There was a sustained and large increase in both the incidence rate and the mortality rate of prostate cancer with an APC of 3.8 and 3.7 respectively.

Breast cancer had an incidence and mortality rates that increased significantly (APC 1.9 and 2.7), however in the last three quinquennial periods the mortality rates remained stable.

There was a significant downward trend in the incidence rates of cervical cancer (APC 2.6). The mortality rates, analyzed globally, did not show a significant decrease.

The incidence rates for thyroid cancer have large differences in magnitude between Men and women which accentuated with the passing of time. Initially, a ratio of 1:2 was detected and in the final years the ratio was 1:5. The incidence rates increased in both men and women, especially in the last two quinquenial. In the case of women, this increase was significant (APC 8.5).

Mortality rates in both men and women remained low, with small variations that were not significant.

The incidence and mortality rates of lymphomas were slightly higher in men. Among men, incidence rates increased over time significantly (APC 2.2). Mortality rates increase in both genders (APC 5.4 men APC 5.8, women).

The incidence rate of leukemia was higher among men. It also increased significantly among women (APC 1.1). On the other hand, mortality had a significant tendency to rise among men (APC 2.6).

## Discussion

The incidence rate in certain body locations is reflected by the demographic, social and economic change that is occurring in Quito and Ecuadorian society. It is essential to highlight the importance of the cancer active registry that has been kept by the RNT over the last three decades. This has allowed identifying the trends of both incidence and mortality of the main types of cancer in the city of Quito ^6^.

While breast cancer is on the increase among women in Quito, cervical cancer on the other hand is decreasing. This behavior indicates the important changes in the lifestyle that women have had, especially in the urban area and in the cities of greater economic development. The inclusion of women in the national economy has led to an increase in the "estrogen window of risk" due to late pregnancies, fewer pregnancies, reduced lactation or caloric overload ^7^. On the other hand, the improved access to health services ^8^, a better educational level of women in the city ^9^, and campaigns of prevention, are changes that should have an impact on the decrease in the incidence of cancer of the cervix. The same tendency occurs more slowly in other places within the country due to sanitary, educational and life style changes. 

Thyroid cancer, which is much more frequent in women, is notable for its tendency to increase. Comparing the increase of incidence from 10 to 35 over the last ten years places this area among one of the highest positions in the world ^10^. 

This increase is due to papillary cancer which represents 44% of the cases (1985-1988) and 89% between the period 2009-2013. Whereas in men, the increase of these rates was moderate and also given in papillary cancer (from 40% to 84% in the same periods).

One explanation for this phenomenon is over diagnosis established through different researches in various countries throughout the world ^11^. Increased medical surveillance and the introduction of new diagnostic techniques, such as neck ultrasound (since the 1980s) and, more recently, computed tomography (CT) and magnetic resonance imaging (MRI), have made it possible to detect a large number of asymptomatic and non-lethal diseases that exist in abundance in the thyroid gland of healthy people of all ages. On the other hand, little is known about the aetiology of thyroid cancer so far; exposure to ionizing radiation (especially during childhood) and a history of benign thyroid disease are the only well-established risk factors for differentiated thyroid carcinomas (the most common forms of thyroid cancer) ^12^ ^-^ ^14^. Based on this data, IARC warns against the systematic detection of the thyroid gland cancer and the study of small nodules, and suggests careful monitoring for patients affected by low-risk tumors.

Gastric cancer decreased in incidence and mortality in both men and women. Worldwide, *Helicobacter pylori* infection is recognized as the primary cause of gastric cancer ^15^. Several risk factors have also been identified, such as socioeconomic level, high-salt and low-antioxidant diet, alcohol and, tobacco consumption ^16^ ^-^ ^18^. However, in Ecuador there are multiple geographical, physical, biological, social, economic and cultural variables which could be related to the occurrence and course of the disease. The equatorial location, the ethnic diversity, multiculturalism, the phenotype of miscegenation and the limited access to education and health services are issues that must be addressed in order to better understand the gastric cancer disease in our country. The decrease, both in incidence and in mortality, is most probably linked to better socioeconomic conditions, improvement of the quality of food and its preservation ^19^, rather than to the intervention of health services in the early diagnosis.

The significant increase in incidence and mortality of colorectal cancer is probably a manifestation of changes in the lifestyles of societies. It is known worldwide that its incidence is higher in countries in economic transition, especially those that have adopted lifestyles typical of industrialized countries (diets with a low intake of fruits and vegetables, greater consumption of red or processed meat, physical inactivity, smoking and alcohol consumption) ^20^.

In men, the great increase in prostate cancer incidence rates is associated with an improvement in early diagnosis, mainly due to the use of prostate antigen. It would be expected that this very important increase is accompanied by a decrease in the mortality rate, however, as has been observed in other countries ^21^, there was no impact on the decrease in mortality.

In women, the incidence and mortality rates of lung cancer have increased significantly. But in men, little changes were observed in their rates. This behavior is probably due to the changes that occurred in the smoking behavior of both sexes. The application of the WHO Framework Convention for the Control of Tobacco (FCTC) and its Protocol for the Elimination of Illicit Trade in Ecuador, makes it possible to expand measures to avoid the risks caused by tobacco and reduce consumption. In this regard, the information provided represents a baseline for evaluating the performance and impact of these policies adopted in 2015, the same year that protocol was ratified in Ecuador ^22^.

In the Ecuadorian constitution of 2008, health was established as a guaranteed right by the State. The health services are an intermediary determinant of health inequities ^23^, due to this fact a reorganisation of the public care system Network and a Complementary Health Network ^24^ was proposed through the Integral Health Care Model in Families and Communities ^25^. The model´s objective is the strengthening of Primary Health Care (Renewed Health, APS-R) as a basis for the organization and functioning of the National Health System. The use of this model is to ensure universal access to quality, equitable, free and efficient health services by the population. SOLCA was created as part of the complementary health network to be a private non-profit institution, which is financed through the sale of Social Security Services and by substantial contributions by the Ministry of Public Health which has had a very important role in dealing with cancer in Ecuador. In this regard, SOLCA attended 31% of the oncological cases of the country for the period 2009-2013, the public sector attended almost 45% and the private sector around 24%. This distribution will improve as the consolidation of the Integral Public Network is reached.

One of the limitations, specific to the collection of information on deaths is the fact that we can mention the percentage of deaths due to poorly defined or ignored causes (ICD 10: R00-R99). In Ecuador during the period 2000-2004 it was 13%, in 2005-2009 it was 11% and in 2010-2014 it was 9%. In the specific case of the RNT of Quito, the histological verification in men and women increased from 71.0% to 87.0%, in the quinquennial periods 1986-1990 to 2006-2010.

Finally, it is worth mentioning that during these three decades of monitoring, the RNT has provided accurate and relevant information for the education of health professionals. It has contributed to designing and establishing programs for the prevention and control of cancer. In addition, it has trained individuals in the implementation and development of other cancer registries in the country that use the same standardized RNT methodology. Actually, there are registries in Quito, Guayaquil, Manabí, Cuenca, Loja and Machala. The information of the RNT has been included in the publication of the IACR "Cancer Incidence in Five Continents", (Volumes VI, VII, VIII, IX, X and XI).
