# Supplementary material for: Trends in cancer incidence and mortality over three decades in Quito - Ecuador
Source: Colomb Med (Cali). 2018 Mar 30;49(1):35–41. doi: 10.25100/cm.v49i1.3785 (PMC6018821; doi:10.25100/cm.v49i1.3785)
Supplement: Related texts

# Research article: Tendencias en incidencia y mortalidad por cáncer durante tres decadas en Quito - Ecuador

## Introducción

La experiencia y consolidación de los Registros poblacionales de cáncer tienen su inicio en Hamburgo Alemania en 1929 ^1^. Su mayor impulso se produjo a partir de la Conferencia de Copenhague en 1946, en la que se recomendó el establecimiento de los Registros de Cáncer a nivel mundial. En 1966 se creó la Asociación Internacional de Registros de Cáncer (IACR por sus siglas en ingles), como parte de la Agencia Internacional de Investigación en Cáncer (IARC por sus siglas en ingles), entidad que aglutina, apoya y establece las directrices para el desarrollo e implementación de los registros de cáncer poblacionales.

En América Latina surgieron los primeros registros de cáncer en la década de los 50 ^2^, el primero fue el de Puerto Rico y en 1962 inició su trabajo el registro de Cali - Colombia. Este último se constituyó en referente y estímulo para consolidar la propuesta en Ecuador.

En 1984, al interior de la Sociedad de Lucha contra el Cáncer (SOLCA) Núcleo Quito, se creó el Registro Nacional de Tumores (RNT) ^3^, que ha recolectado, procesado, analizado y divulgado regularmente la información de los casos nuevos de cáncer diagnosticados en la ciudad de Quito. El presente estudio analizó la tendencia de la incidencia y mortalidad de los principales tipos de cáncer en la ciudad de Quito, desde 1985 hasta el año 2013.

## Materiales y Métodos

El RNT capta todos los casos de cáncer diagnosticados en la ciudad de Quito. La metodología es la adoptada internacionalmente (IARC - IACR). La información se obtiene mediante un proceso activo, en el que técnicos adscritos al registro de cáncer acuden a los laboratorios de patología, hematología y citología de todos los establecimientos y consultas de salud pública y privada de la ciudad de Quito. Revisan los archivos para detectar casos diagnosticados de cáncer y en un segundo momento revisan la historia clínica o establecen contacto con el médico tratante para obtener mayor información acerca del paciente. La metodología garantiza que no se produzca duplicidad de casos.

Los casos en que nunca se realizó un examen microscópico se los captó en el Instituto Nacional de Estadística y Censos (INEC) a través de la revisión de “egresos hospitalarios” y en las “defunciones por cáncer” ocurridas en la ciudad de Quito, que actualmente constituyen cerca del 6% de los casos nuevos de cáncer. Una limitación de los egresos hospitalarios es no discriminar en forma adecuada las múltiples ocasiones que un mismo paciente ingresa y egresa de un hospital o, cánceres que no requieren hospitalización.

Los egresos y las defunciones fueron confrontados con la base de datos del Registro, con el objetivo de identificar casos nuevos o actualizar el estado vital de los pacientes previamente registrados.

Las variables que se recolectaron fueron organizadas en tres áreas: identificación del paciente, descripción del tumor y extensión clínica del tumor. La estadificación se realizó para las ocho localizaciones más frecuentes (cérvix, mama, próstata, pulmón, colon-recto, estómago, tiroides y linfomas).

Se consideró un caso, a toda neoplasia maligna invasiva o *in situ*, incidente en el año, con o sin verificación histológica, ocurrida en la población residente de la ciudad de Quito. 

No se registraron los tumores de naturaleza incierta. Desde el 2005 se recaudó y procesó con la Clasificación CIEO3. Se registraron tumores, no pacientes. La definición de tumor múltiple se estableció con los criterios definidos por la IARC ^4^.

La información recolectada fue validada a través de controles de calidad cruzados entre los técnicos y resolución de dudas con los médicos del Registro y/o el apoyo ocasional de un médico patólogo. Al finalizar cada año se aplicó la herramienta de validación: IARCcrgTools 2.05, para establecer la coherencia de las principales variables que debe tener un Registro poblacional. 

El RNT cuenta con un sistema informático que está en su cuarta versión y se ajusta a las necesidades de la metodología implementada. Los datos se presentan de acuerdo a la Clasificación Internacional de Enfermedades CIE 10, y se analizaron por sexo, las tasas de incidencia y mortalidad estandarizadas por edad (población estándar mundial, método directo), en seis períodos: quinquenales 1985-1988, 1989-1993, 1994-1998, 1999-2003, 2004-2008 y 2009-2013, en las localizaciones seleccionadas. Para estudiar la tendencia de las tasas de incidencia y mortalidad por cáncer durante un período 1985-2013 se estimó el cambio porcentual anual promedio (CPA). Al describir el cambio, los términos ‘’aumenta’’ o ‘’disminuye’’ se usaron cuando el CPA fue significativamente diferente de cero (valores de *p* <0.05); de lo contrario, se utilizó el término “estable”. Las pruebas de significancia se realizaron usando la técnica de permutación de Monte Carlo. Todos los análisis se realizaron con el Programa de Regresión Joinpoint versión 4.5.0.1, del Programa de Investigación de Vigilancia del Instituto Nacional del Cáncer de los Estados Unidos.

### Población

La ciudad de Quito es la capital del Ecuador, es la segunda ciudad más grande y poblada del país. Se encuentra en la latitud 0°13’23” Sur, al occidente de la Cordillera de los Andes, a 2,800 metros sobre el nivel del mar. La extensión de la ciudad es de 127 Km^2^ y su población para el año 2013, de acuerdo a las proyecciones del Censo del año 2010 es: 1,694,086 habitantes ^5^. 

La población del Ecuador en general y de Quito en particular, ha experimentado cambios importantes en su composición etárea y velocidad de crecimiento. El Ecuador, en las tres últimas décadas, ha desarrollado censos en los años 1982, 1990, 2001 y 2010. A través de sus datos se puede observar que la base ancha de su pirámide poblacional disminuye en tanto que el grupo de mayor edad se incrementó. En Quito en 1985 el 54% era menor de 25 años en tanto que en el 2013 este grupo se redujo al 44%. Los de 65 años y más representaron 4.3% en 1985 y en el 2013, 6.5% (Fig. 1). Los datos poblacionales de los años intercensales son proporcionados por el INEC. 

**Figura 1:** Estructura Poblacional de Quito por edad y sexo: 1985 - 2013.

## Resultados 

En la Tabla 1 se presentan las tasas de incidencia estandarizadas por 100.000 habitantes según sexo, de las principales localizaciones y en seis períodos. Se presentan también las tendencias de la incidencia y su intervalo de confianza, los valores del cambio porcentual anual y la significación estadística. 

**Tabla 1:** Quito, Ecuador. Incidencia. Tasas estandarizadas por Edad para Localizaciones seleccionadas por sexo. 1985 - 2013

Localización	Tasas de Incidencia estandarizadas por edad (Población Mundial) por períodos												Tendencia de la tasa de incidencia			
	1985-1988		1989-1993		1994-1998		1999-2003		2004-2008		2009-2013		Hombres		Mujeres	
	♂	♀	♂	♀	♂	♀	♂	♀	♂	♀	♂	♀	CPA	IC 95%	CPA	IC 95%
Labio Cav Oral Faringe	2.0	1.6	1.8	1.1	2.4	1.7	1.7	1.3	1.9	2.0	2.1	1.6	0.0	-1.2; 1.3	0.9	-1.4; 3.2
Esófago	3.4	0.6	3.0	1.2	2.4	0.6	2.0	0.8	2.4	0.6	1.4	0.7	-3.1*	-4.6; -1.5	~	
Estómago	28.5	21.7	32.7	20.0	24.5	16.9	22.2	13.2	22.9	14.9	20.3	14.5	-1.7	-5.3; 2.1	-1.6*	-2.7; -0.5
Colon recto	7.3	8.2	7.4	9.0	8.7	8.1	8.9	8.7	11.3	10.2	13.2	11.9	2.5*	1.7; 3.3	1.4*	0.6; 2.2
Páncreas	4.4	3.6	3.7	4.2	3.8	3.5	3.2	3.1	2.8	3.6	3.2	3.9	-1.3*	-2.3; -0.3	0.2	-1.2; 1.5
Laringe	2.0	0.2	1.3	0.2	1.5	0.1	1.3	0.1	1.5	0.2	1.6	0.2	-0.1	-1.9; 1.7	~	
Bronquios Pulmón	7.9	3.3	10.1	3.7	8.3	4.8	8.4	4.4	8.0	5.8	8.0	6.4	-0.5	-1.3; 0.4	2.8*	1.7; 3.9
Cérvix	-	31.0	-	32.6	-	24.8	-	19.5	-	18.6	-	18.6	-	-	-2.6*	-3.2; -2.0
Cuerpo Uterino	-	4.8	-	5.3	-	4.5	-	4.4	-	4.4	-	5.6	-	-	0.3	-0.6; 1.3
Mama	-	25.4	-	26.6	-	28.4	-	31.4	-	36.8	-	38.8	-	-	1.9*	1.4; 2.3
Ovario	-	5.7	-	6.2	-	7.5	-	6.5	-	7.8	-	7.5	-	-	1.2*	0.4; 2.0
Próstata	22.7	-	23.1	-	31.7	-	43.5	-	53.5	-	62.9	-	3.8*	2.2; 5.3	-	-
Testículo	2.6	-	4.0	-	3.3	-	4.2	-	5.2	-	5.7	-	3.0*	2.0; 4.1	-	-
Vejiga	4.3	2.0	5.9	1.4	5.0	1.3	5.3	1.5	5.1	2.0	6.1	2.2	0.8	-0.3; 1.9	1.4	-0.2; 3.0
Tiroides	3.1	6.3	2.1	7.6	2.2	8.7	2.4	10.5	3.9	19.6	6.6	35.0	3.6	-0.6; 8.1	8.5*	5.6; 11.5
Linfoma	8.9	7.2	8.8	7.5	11.4	8.4	10.5	9.1	12.6	9.9	16.1	13.1	2.2*	1.4; 3.1	1.7	-1.9; 5.3
Leucemia	7.1	5.5	6.7	5.2	8.4	7.2	7.7	6.2	7.7	6.6	8.6	7.0	0.8	-0.1; 1.8	1.1*	0.2: 2.1
Melanoma	2.3	3.3	3.5	3.6	2.2	3.0	2.5	3.0	3.7	3.8	4.1	3.8	1.8*	0.2; 3.4	1.2	-0.3; 2.7
Todos - piel no melan	142.3	185.9	146.8	190.1	147.6	177.3	158.5	167.2	182.1	201.2	200.8	207.9	1.4*	0.9; 2.0	1.0*	0.5; 1.5

Tasas x 100,000

CPA: cambio porcentual anual promedio. * El CPA fue significativamente diferente de cero (*p* <0.05)

~ No fue posible calcular

En la Figura 2 podemos observar la tendencia en la incidencia y la mortalidad en las principales localizaciones del cáncer. En hombres, se observó un incremento significativo de la tasa de incidencia global, con un CPA de 1.4% (Fig 2a). La tasa de incidencia de cáncer de estómago fue la más alta en los dos primeros períodos, superada luego por el cáncer de próstata. 

**Figure 2:** Quito, Ecuador. **a.** Tasas de incidencia de cáncer. 1985 - 2013 (Localizaciones seleccionadas). Hombres b. Tasas de incidencia de cáncer. 1985 - 2013 (Localizaciones seleccionadas). Mujeres. **c.** Tasas de incidencia y mortalidad por cáncer. 1985 - 2013.

En mujeres, la tasa de incidencia global también se incrementó en forma significativa con un CPA de 1% (Fig 2b). En los dos primeros períodos el cáncer más frecuente fue el de cuello uterino y en los siguientes períodos se presentó el cáncer de mama con la tasa más alta. En los dos últimos períodos apareció el cáncer de tiroides en segundo lugar, superando al cáncer de cuello uterino.

La Tabla 2 presenta las tasas estandarizadas de mortalidad por cáncer en hombres y mujeres en los seis períodos. En hombres, el cáncer que más muertes causó hasta el período 1994-1998, fue el de estómago. En los tres últimos quinquenios la tasa más alta de mortalidad fue causada por el cáncer de próstata. A nivel nacional la situación fue diferente en cuanto a la mortalidad, el cáncer de estómago hasta el año 2013 fue la primera causa de muerte entre los tumores malignos. 

**Tabla 2:** Quito, Ecuador. Tasas de mortalidad por cáncer estandarizadas por edad según sexo.1982-2013.

Localización	Tasas de mortalidad estandarizadas por edad (Población Mundial)												Tendencia de la tasa de mortalidad			
	1985-1988			1989-1993		1994-1998		1999-2003		2004-2008		2009-2013		Hombres		Mujeres	
	♂	♀	♂	♀	♂	♀	♂	♀	♂	♀	♂	♀	CPA	IC 95%	CPA	IC 95%
Labio Cav Oral Faringe	0.8	0.5	0.8	0.3	0.8	1.0	1.0	0.8	1.0	0.7	1.3	0.8	2.4*	0.1; 4.7	~	
Esófago	2.6	0.3	2.0	0.6	1.8	0.4	1.7	0.4	1.4	0.5	1.3	0.6	-2.3*	-3.8; -0.7	~	
Estómago	16.7	13.8	18.4	11.5	16.2	11.6	15.8	10.2	15.9	10.6	15.2	10.0	-0.5*	-0.9; -0.1	-0.9*	-1.7; -0.1
Colon recto	3.3	2.9	3.5	3.8	4.4	4.9	5.5	4.7	5.9	5.5	7.0	6.4	3.5*	2.4; 4.6	4.2*	0.3; 8.2
Páncreas	2.7	2.6	2.8	3.3	3.1	2.7	2.5	2.5	2.5	3.3	2.6	3.1	-0.1	-1.3; 1.1	0.6	-0.6; 1.9
Laringe	1.0	0.0	0.4	0.2	0.4	0.1	0.7	0.0	1.0	0.0	0.9	0.1	~		~	
Bronquios Pulmón	5.2	2.8	7.2	2.9	6.7	3.5	7.0	3.6	6.7	4.6	6.2	4.9	0.4	-0.6; 1.4	2.7*	1.6; 3.7
Cérvix	-	8.6	-	10.7	-	10.5	-	10.2	-	10.3	-	8.2	-	-	0.1	-5.3; 5.8
Cuerpo Uterino	-	0.4	-	1.2	-	1.5	-	1.4	-	1.6	-	1.6	-	-	~	
Mama	-	6.5	-	7.8	-	9.6	-	12.1	-	12.4	-	12.3	-	-	2.7*	1.5; 4.0
Ovario	-	2.2	-	2.4	-	4.1	-	3.5	-	4.0	-	4.2	-	-	2.7*	1.3; 4.0
Próstata	9.3	-	10.8	-	12.7	-	16.3	-	18.7	-	1.9-1.9	-	3.7*	3.0; 4.4	-	-
Testículo	0.6	-	0.7	-	0.6	-	08	-	0.9	-	0.7	-	1.1	-1.4; 3.8	-	-
Vejiga	1.0	0.5	1.9	0.6	1.8	0.6	27	1.0	2.4	0.7	2.6	1.2	5.7*	1.0; 10.5	2.9*	0.1; 5.7
Tiroides	1.1	1.4	0.9	1.7	0.9	1.2	08	1.3	0.9	2.0	0.9	2.2	0.3	-2.2; 2.8	1.8	-1.5; 5.2
Linfoma	2.0	1.8	3.3	2.2	5.2	3.7	54	4.6	6.9	5.2	7.9	5.9	5.4*	3.9; 6.9	5.8*	2.7; 9.0
Leucemia	3.3	2.3	3.0	2.0	4.7	4.3	52	3.6	5.3	4.1	5.2	3.9	2.6*	1.5; 3.8	2.2	-3.5; 8.1
Melanoma	0.3	0.7	0.8	0.6	1.0	0.9	11	0.9	1.4	1.4	2.0	1.5	~		4.7*	2.0; 7.5
Todos - piel No melan	68.4	69.0	73.0	75.1	78.9	83.6	88.1	83.1	93.7	90.3	101.2	89.4	1.7*	1.4; 2.0	1.2*	0.8; 1.5

Tasas x 100,000

CPA: cambio porcentual anual promedio. * El CPA fue significativamente diferente de cero (*p* <0.05)

~ No fue posible calcular

En mujeres residentes en Quito, el comportamiento de la mortalidad por cáncer gástrico fue similar al de los hombres con dos características: a) las tasas presentaron valores inferiores y b) en el período 1999-2003 el cáncer de mama sobrepasó al de estómago como causa de muerte.

Las tasas de incidencia de cáncer gástrico disminuyeron en las mujeres en forma significativa con CPA de -1.6. En hombres la disminución global no fue significativa. Sin embargo, al aplicar la regresión joinpoint se observó una primera fase de incremento y a partir del período 1994-1998 hasta el 2013, la disminución fue significativa. (CPA -2.4, IC95%: -3.8; -1.1) Las tasas de mortalidad decrecieron de manera significativa, en hombres y mujeres (-0.5 y -0.9, respectivamente) (Fig. 2c).

El cáncer colon-rectal tuvo una tendencia al ascenso en forma significativa tanto en hombres (CPA de 2.5), como en mujeres, (CPA 1.4). Los valores fueron muy similares para los dos sexos. Las tasas de mortalidad, en hombres y mujeres fueron crecientes de manera significativa (3.5 y 4.2, respectivamente)

En los 29 años de análisis, la incidencia y la mortalidad por cáncer de pulmón, entre los hombres, se mantuvieron estables. En las mujeres, el incremento fue significativo con CPA de 2.8 en incidencia y 2.7 en mortalidad. Se apreció un incremento sostenido y de gran magnitud tanto de la tasa de incidencia como la de mortalidad de cáncer de próstata con un CPA de 3.8 y 3.7 respectivamente. 

El cáncer de mama presentó tasas de incidencia y mortalidad que se incrementaron en forma significativa (CPA 1.9 y 2.7), sin embargo en los tres últimos quinquenios las tasas de mortalidad se mantuvieron estables. 

Se observó una tendencia descendente significativa de las tasas de incidencia del cáncer de cérvix (CPA -2.6). Las tasas de mortalidad, analizadas globalmente, no mostraron una disminución significativa. 

Las tasas de incidencia de cáncer de tiroides tuvieron grandes diferencias en magnitud entre hombres y mujeres, que se acentuaron en el tiempo. Inicialmente se observó una relación de 1:2 y en el último la relación fue de 1:5. Las tasas de incidencia se incrementaron tanto en hombres, como en mujeres, especialmente en los dos últimos quinquenios. Para el caso de las mujeres este incremento fue significativo (CPA 8.5). Las tasas de mortalidad tanto en hombres como en mujeres se mantuvieron bajas, y las pequeñas variaciones no fueron significativas. 

Las tasas de incidencia y mortalidad de linfomas fueron ligeramente mayores en hombres. Entre los hombres, las tasas de incidencia se incrementaron en forma significativa (CPA 2.2). Las tasas de mortalidad se incrementaron en ambos sexos (CPA 5.4 hombres CPA 5.8, mujeres).

La tasa de incidencia de leucemias fue mayor entre los hombres. Se incrementó en forma significativa entre las mujeres (CPA 1.1). Por otra parte, la mortalidad tuvo una tendencia significativa al ascenso entre los hombres (CPA 2.6) .

## Discusión 

Las tasas de incidencia en ciertas localizaciones del cuerpo, se reflejan en los cambios demográficos, sociales y económicos que están ocurriendo en la sociedad quiteña y ecuatoriana. Es fundamental destacar la importancia del registro activo de cáncer que ha mantenido el RNT durante las últimas tres décadas. Esto ha permitido mostrar con mayor exactitud la tendencia de incidencia y mortalidad de los principales tipos de cáncer en la ciudad de Quito ^6^.

Mientras el cáncer de mama se está incrementado entre las mujeres de Quito, el cáncer de cuello uterino está disminuyendo. Este comportamiento expresa los importantes cambios en el estilo de vida que han tenido las mujeres, especialmente en el área urbana y en las ciudades de mayor desarrollo económico. La inclusión de la mujer en la economía nacional ha determinado un aumento de la “ventana estrogénica de riesgo” ya sea por embarazos tardíos, menor número de embarazos, disminución de la lactancia o sobrecarga calórica ^7^. Por otro lado, el mayor acceso a los servicios de salud ^8^, el mejor nivel educativo de las mujeres en la ciudad ^9^, y las campañas informativas de prevención, son cambios que debieron impactar en la disminución de la incidencia del cáncer de cérvix. Esta misma tendencia ocurre de manera más lenta en otras localidades del país, debido a la incorporación de los cambios sanitarios, educativos y estilos de vida. 

El cáncer de tiroides mucho más frecuente en mujeres, llama la atención por su tendencia al incremento. Comparando el incremento desde 10 hasta 35 ocurrido en los últimos 10 años nos ubica entre las más altas del mundo ^10^. Este incremento fue a expensas del cáncer papilar que constituye el 44% de la casuística entre 1985-1988 y el 89% entre 2009-2013. En hombres el incremento de tasas fue moderado y también a expensas del papilar (del 40% al 84% entre los mismos períodos).

Una explicación para la comprensión de este fenómeno es el sobrediagnostico establecido por diferentes investigaciones en diferentes países en el mundo ^11^. La creciente vigilancia médica y la introducción de nuevas técnicas de diagnóstico, como la ecografía de cuello (desde la década de 1980) y, más recientemente, la tomografía computarizada (TC) y la resonancia magnética (IRM), han permitido detectar un gran número de enfermedades asintomaticas y no letales que existen en abundancia en la glándula tiroides de personas sanas de cualquier edad. Por otro lado, poco se sabe sobre la etiología del cáncer de tiroides hasta el momento; la exposición a la radiación ionizante (especialmente durante la infancia) y los antecedentes de enfermedad tiroidea benigna son los únicos factores de riesgo bien establecidos para los carcinomas diferenciados de tiroides (las formas más comunes de cáncer de tiroides) ^12^^-^^14^. Con base en estos datos, la IARC advierte contra la detección sistemática del cáncer de la glándula tiroides y el estudio de pequeños nódulos, sugiere una monitorización cuidadosa para los pacientes afectados por tumores de bajo riesgo.

El cáncer gástrico decreció en incidencia y mortalidad tanto en hombres como en mujeres. Mundialmente se reconoce a la infección por *Helicobacter pylori* como la causa primaria del cáncer gástrico ^15^, se han identificado también varios factores de riesgo como el nivel socioeconómico, la dieta alta en sal y baja en antioxidantes y el consumo de alcohol y tabaco ^16^^-^^18^. Sin embargo, en Ecuador confluyen múltiples variables geográficas, físicas, biológicas, pero también sociales, económicas y culturales que podrían estar relacionadas con la ocurrencia y el curso de la enfermedad. La ubicación ecuatorial, la plurietnia, la multicultura, el fenotipo propio del mestizaje y el acceso limitado a la educación y a los servicios de salud son temas que deben ser abordados para una mayor comprensión del problema en el país. La disminución, tanto en incidencia como en mortalidad, probablemente está relacionada a las mejores condiciones socioeconómicas, mejoramiento de la calidad de la alimentación y de su preservación ^19^, antes que a la intervención de los servicios de salud en el diagnóstico temprano.

El incremento significativo en incidencia y en mortalidad del cáncer colorectal probablemente es una manifestación de los cambios de estilos de vida de las sociedades. Mundialmente se conoce que su incidencia es alta en países en transición económica, especialmente en aquellos que han adoptado estilos de vida típicos de países industrializados (dietas con una baja ingesta de frutas y verduras, mayor consumo de carne roja o procesada, inactividad física, tabaquismo y consumo de alcohol) ^20^.

En hombres el gran aumento de las tasas de incidencia de cáncer de próstata se vincula con una mejora en el diagnóstico temprano debido fundamentalmente, al uso del antígeno prostático. Se esperaría que este importantísimo incremento se acompañe de una disminución de la tasa de mortalidad, sin embargo, como se ha observado en otros paises ^21^, no hubo un impacto en la disminución de la mortalidad. 

En mujeres, las tasas de incidencia y mortalidad de cáncer de pulmón tuvieron un crecimiento significativo. En hombres se observó estabilidad de las tasas. Este comportamiento probablemente se deba a los cambios que se han dado por el uso de tabaco en ambos sexos. La aplicación del Convenio Marco de la OMS para el Control del Tabaco (CMCT) y su Protocolo para la Eliminación del Comercio Ilícito en Ecuador, posibilita ampliar las medidas para evitar los riesgos que ocasiona el tabaco y reducir el consumo. En ese sentido, la información aportada representa una línea de base para evaluar el desempeño e impacto de las políticas tomadas a partir del año 2015, año en que se ratificó el protocolo en Ecuador ^22^.

En la constitución ecuatoriana del año 2008 se estableció a la salud como un derecho garantizado por el Estado. Los servicios de salud son un determinante intermediario de las inequidades en salud ^23^, por este motivo, se planteó reorganizar los sistemas de atención en una Red Pública y una Red Complementaria de Salud ^24^ a través del Modelo de Atención Integral en Salud Familiar y Comunitario ^25^. Esta modelo está dirigido a fortalecer la Atención Primaria en Salud Renovada (APS-R) como base de la organización y funcionamiento del Sistema Nacional de Salud y así concretar su objetivo de asegurar un acceso universal a la población a los servicios de salud con calidad, equitativos, gratuitos y eficientes. SOLCA, como parte de la red complementaria de salud, al ser una institución privada sin ánimo de lucro, que se financia a través de la venta de Servicios a la Seguridad Social y al Ministerio de Salud Pública, aporta al manejo de los casos de cáncer en Ecuador. Al respecto, SOLCA atendió al 40% de los casos oncológicos de Quito, durante los años 2009-2013, mientras que el sector público incluida la Seguridad Social, atendió el 38% y el sector privado con fines de lucro al 22%. Esta distribución irá cambiando en la medida que se consolide la construcción de la Red Pública Integral.

Como una limitante de este estudio en la recolección de la información sobre defunciones, podemos mencionar el porcentaje de defunciones por causas mal definidas o ignoradas (CIE 10: R00-R99). En el Ecuador durante el período 2000-2004 fue de 13%, en el 2005-2009 de 11% y en el 2010-2014 del 9%. En el caso concreto del RNT de Quito, la verificación histológica en hombres y mujeres aumentó de 71% a 91% desde el quinquenio 1986-1990 al 2009 -2013.

Finalmente, vale la pena mencionar que durante estas tres décadas de monitoreo, el RNT ha proporcionado información precisa y relevante para la educación de profesionales de la salud. Ha aportado a diseñar y establecer programas para la prevención y el control del cáncer. Además ha capacitado en la implementación y desarrollo de otros registros de cáncer en el país que funcionan con la misma metodología del RNT. Al momento existen registros en Quito, Guayaquil, Manabí, Cuenca, Loja y Machala. Los datos del RNT han sido incorporados, desde sus inicios, en la publicación de la IACR “Cancer Incidence in Five Continents”, (Volúmenes VI, VII, VIII, IX, X y XI).
